# Disparities in length of stay for hip fracture treatment between patients treated in safety-net and non-safety-net hospitals

**DOI:** 10.1186/s12913-020-4896-1

**Published:** 2020-02-10

**Authors:** Edward Coffield, Saeyoan Thirunavukkarasu, Emily Ho, Swapna Munnangi, L.D. George Angus

**Affiliations:** 1 Department of Health Professions, Hofstra University, 262 Swim Center, 220 Hofstra University, Hempstead, NY 11549-2200 USA; 2 Department of Data Analytics, Alliance for Positive Change, 64 West 35th Street, New York, NY 10001 USA; 3 Department of Surgery, Nassau University Medical Center, 2201 Hempstead Turnpike, East Meadow, NY 11554 USA

**Keywords:** Health care disparities, Length of stay, Safety-net hospital, Hip fracture, Quality of care

## Abstract

**Background:**

Length of hospital stay (LOS) for hip fracture treatments is associated with mortality. In addition to patient demographic and clinical factors, hospital and payer type may also influence LOS, and thus mortality, among hip fracture patients; accordingly, outcome disparities between groups may arise from where patients are treated and from their health insurance type. The purpose of this study was to examine if where hip fracture patients are treated and how they pay for their care is associated with outcome disparities between patient groups. Specifically, we examined whether LOS differed between patients treated at safety-net and non-safety-net hospitals and whether LOS was associated with patients’ insurance type within each hospital category.

**Methods:**

A sample of 48,948 hip fracture patients was extracted from New York State’s Statewide Planning and Research Cooperative System (SPARCS), 2014–2016. Using means comparison and X^2^ tests, differences between safety-net and non-safety-net hospitals on LOS and patient characteristics were examined. Relationships between LOS and hospital category (safety-net or non-safety-net) and LOS and insurance type were further evaluated through negative binomial regression models.

**Results:**

LOS was statistically (*p* ≤ 0.001) longer in safety-net hospitals (7.37 days) relative to non-safety-net hospitals (6.34 days). Treatment in a safety-net hospital was associated with a LOS that was 11.7% (*p* = 0.003) longer than in a non-safety-net hospital. Having Medicaid was associated with a longer LOS relative to having commercial health insurance.

**Conclusion:**

Where hip fracture patients are treated is associated with LOS and may influence outcome disparities between groups. Future research should examine whether outcome differences between safety-net and non-safety-net hospitals are associated with resource availability and hospital payer mix.

## Background

Within the United States patients treated for hip fractures are estimated to have a one-year mortality rate between 8.4 and 36.0% [1]. Patient comorbidities [2–6], demographic characteristics [7–9], and support networks or living arrangements upon discharge [5, 10, 11] have all been found to influence hip fracture treatment outcomes. These outcomes, however, are not homogenous across the population; researchers have identified differences in hip fracture treatment outcomes among racial and socioeconomic groups [12]. Disparities between groups on health care outcomes may arise from patient-level factors [13, 14] as well as from where patients receive treatment [15–17] and their health insurance status [13, 14, 18]. The primary objective of this study was to examine whether hip fracture treatment outcomes differed between patients who received care at safety-net hospitals (e.g., hospitals with a payer mix consisting of a large share of Medicaid and uninsured patients) and patients who received care at non-safety-net hospitals.

Safety-net hospitals (SNH) by definition provide a large amount of their services to uninsured persons, people with Medicaid health insurance, and other vulnerable populations [19]. SNH are eligible for Medicaid disproportionate share payments (DSH) to help offset the cost of treating uninsured patients [19]. However, Gilman et al. [20] estimated that SNH have lower margins than non-safety-net-hospitals (NSNH) while Manary et al. [21] found that hospitals’ overall financial health was driven by the percentage of hospitals’ patients who had private health insurance. Similarly, in a systematic review, Oner et al. [22] found, in the only two studies with significant results (out of eight), a negative relationship between hospitals’ total margins and hospitals’ Medicaid payer mix share.

Hospitals’ payer mixes may also influence their investment decisions [21] and quality of care [23]. For instance, having payer mixes with a large portion of Medicaid and self-pay (or uninsured) payers may result in SNH having limited resources to adopt quality improvement measures as rapidly or as frequently as hospitals with lower shares of Medicaid payers and higher shares of commercially insured payers. The variation in payer mix between SNH and NSNH could thus result in differences in the quality of care provided and patient outcomes between SNH and NSNH. Indeed, Hoehn et al. [24] found that SNH had fewer resources, higher readmission and mortality rates, and a higher cost of care relative to other hospitals. Accordingly, the type of hospital (SNH or NSNH) where patients are treated (and the payer mix of the hospital) could help explain health care disparities among different groups of hip fracture patients.

In addition to other hospital types [16, 17], researchers found outcome differences between patients treated at SNH and those treated at NSNH [8, 25–28]. Furthermore, Kumar et al. [29] estimated that hip fracture patient outcomes differed within a skilled nursing facility based upon patients’ health insurance types. However, minimal research has directly examined whether hip fracture patient treatment outcomes differ *between* SNH and NSNH or whether hip fracture patient treatment outcomes differ *within* SNH and NSNH based upon patients' health insurance types.

This study evaluated whether health care outcomes of hip fracture patients, treated in New York State from 2014 to 2016, differed according to where they were treated and their health insurance types. Specifically, the study examined two questions. First, we evaluated whether hip patient treatment outcomes differed according to whether patients were treated at a SNH or a NSNH (between hospital examination). Second, we estimated whether hip fracture patient outcomes were associated with their health insurance coverage within each hospital type (within hospital examination). The *between* hospital type comparisons provide insight into whether disparities are associated with where hip fracture patients are treated (type I health care disparity [14]). The *within* hospital type comparisons allow an examination into whether patients’ health insurance types are associated with their hip fracture treatment outcomes (type II health care disparity [14]).

Length of stay was used in this study to compare whether patients’ hip fracture treatment outcomes differed according to where they were treated and their health insurance type. Length of stay (LOS) may be considered a process measure; however, Brasel, Lin, Nirula et al. argue that focusing on process and structure could result in better outcomes [30]. Furthermore, while two European-based studies did not find a significant relationship between LOS for hip fracture treatments and mortality [31, 32], a New York State-based study illustrated that shorter LOS for hip fracture patients were associated with lower mortality risk [33]. LOS for hip fracture treatments may differ between SNH and NSNH due to variation in treatment protocols, post-care management, and other hospital specific factors [8].

Examining whether LOS for hip fracture treatment varies between SNH and NSNH is important from a quality and equity perspective. If differences exist, future research could investigate specific hospital-based factors (e.g., equipment available, differences in care management protocols) and whether a relationship between these factors and the payer mix of hospitals may help explain the gap between SNH and NSNH. Furthermore, with changes in hospital reimbursement (e.g., quality-based metrics) and the possibility of funding changes for SNH arising from the Patient Protection and Affordable Care Act (e.g., changes in disproportionate share funding) [34, 35], whether LOS differences exist between SNH and NSNH merits evaluation. If differences between SNH and NSNH are partially due to payer mix, reducing funding for SNH could further increase disparities between patients treated at SNH relative to patients treated at NSNH [24], including patients treated for hip fractures.

## Methods

The sample population was drawn from New York State’s Statewide Planning and Research Cooperative System (SPARCS) database. SPARCS is a population-based data system where acute and ambulatory care institutions report patient level clinical, demographic, and payer information to the State regarding each patient’s stay [36]. Patients in the SPARCS dataset who received hip fracture treatments from 2014 to 2016, identified using the Clinical Classification Software Diagnosis Code (CCS) of 226, were considered for inclusion in the study. The CCS coding system underwent changes in the move from the ninth to tenth version of the International Classification of Diseases (ICD), which occurred between 2014 and 2015. However, in this study’s sample, admissions for CCS Code 226 were similar between 2014 and 2015 as well as between 2015 and 2016 indicating a consistency in admissions for the code during the changeover from ICD-9 to ICD-10. This consistency supports the use of this analytic time period.

To create the overall study sample, patients who were treated at hospitals with low hip fracture treatment volumes (lower 5.01% of hospitals by hip fracture treatment volume) and who had LOS over 120 days (top coded in SPARCS) (*n* = 12) were excluded from the study. Information extracted from SPARCS included: age group, gender, race, Spanish/Hispanic ethnicity, admission unit, discharge disposition, length of stay, All Patient Refined Diagnostic Related Groups (APR-DRG) severity of illness level [37], APR-DRG risk of mortality level [37], insurance type, and the name of the hospital where the patient was treated. Hospitals where at least 30% of patients had Medicaid or were uninsured were classified as SNH. This SNH definition was motivated by New York State’s Delivery System Reform Incentive Payment Program’s (DSRIP) SNH requirement that at least 30% of a hospital’s inpatient treatments were associated with Medicaid, uninsured, or Dual Eligible payers; a requirement that was one of the three criteria the State used, with exceptions and appeals, to classify hospitals as SNH under DSRIP [38]. In this study, the percentage of hospitals' patients who had Medicaid or were uninsured was calculated using SPARCS payer (i.e., first health insurance type of patients) and hospital information for hip fracture treatments from 2014 to 2016.

The statistical analysis consisted of summary statistic comparisons and negative binominal regression models (NBRM). Summary statistics were calculated for SNH and NSNH; X^2 ^tests and t-tests (adjusted for unequal variances as needed [39]) were used to examine if LOS and patient characteristics differed between SNH and NSNH. LOS was measured as the difference between patients’ discharge dates and admission dates. The first NBRM evaluated whether LOS for hip fracture treatments was associated with the type of hospital where patients were treated (SNH and NSNH) while adjusting for covariates. A NBRM was selected due to the count-based nature of the dependent variable, LOS, and was used instead of a Poisson model as a likelihood ratio test indicated the possible presence of overdispersion (X^2^ = ~ 23,000; *p* < 0.001) during preliminary modeling. The NBRM model was adjusted with the Huber-White modified sandwich estimator [39, 40] to account for the clustered nature of the data (i.e., groups of patients received treatments at the same hospitals), which could result in biased standard errors.

The independent variable of interest in the NBRM was a binary indicator variable denoting whether patients were treated at a SNH. To help isolate the relationship between LOS and SNH, a number of covariates were included in the model. Theory (e.g., injury severity may be associated with LOS) and previous studies examining hip fracture treatment outcomes [7–9, 33] motivated the covariate selection. Model covariates included: age group (in years: 0–17; 18–29; 30–49; 50–69; 70 years of age and over), gender (female, male), Spanish/Hispanic ethnicity, admission type (emergency/trauma/urgent or other), APR-DRG severity of illness level (minor, moderate, major, extreme), APR-DRG risk of mortality level (minor, moderate, major, extreme), first insurance type (commercial, Medicaid, Medicare, Dual Eligible (Medicaid and Medicare: all of a patients’ payment types were used to generate Dual Eligible), self-pay, unknown/other), and discharge disposition (deceased, home, inpatient rehabilitation hospital, other, nursing home/long term care hospital, skilled nursing facility). The age groups used in the model were as listed in the SPARCS dataset. While a continuous age variable is preferable, such a variable was not available within the public-use dataset. It should also be noted that selection bias could be present if patients selected their treatment hospitals; however, the APR-DRG severity of illness and risk of mortality variables may serve to adjust for differences in case-mix and risk profiles in the presence of such bias, partially alleviating this modeling limitation.

NBRMs were also used to examine the second study question: whether LOS was associated with patient health insurance type within each hospital category (SNH, NSNH). For this question, a separate NBRM was estimated for each hospital category. The independent variables of interest in these models were the health insurance types. Additional model covariates were the same as illustrated above, excepting the SNH indicator variable which was excluded from the models. A final NBRM model was estimated to examine whether LOS was associated with being treated at a SNH when the sample was limited to patients 70 years of age and older. This model included all the covariates illustrated above with the exception of the age variables. All analyses were performed in Stata 15 [41]; a *p*-value < 0.05 was used to determine statistical significance.

## Results

The sample consisted of 48,948 patients; 16,310 patients or 33.32% of the sample population were treated at SNH. Statistical differences (*p* < 0.05) were found between SNH and NSNH on a number of patient characteristics in the summary statistic comparisons (Table 1). Notably, the average LOS for hip fracture patients treated at SNH, 7.37 days, was statistically different (*p* < 0.001) from the average LOS of patients treated at NSNH, 6.34 days. It was also estimated that SNH, on average, had larger shares of patients who were under 70 years of age, male, non-White, and of Spanish/Hispanic descent relative to NSNH. Furthermore, SNH had larger shares of patients who were discharged to their home or an inpatient rehabilitation facility and had Medicaid insurance or both Medicaid and Medicare (Dual Eligible) insurance relative to NSNH. Conversely, relative to NSNH, SNH had a smaller share of patients who were discharged to a skilled nursing facility or a long-term care facility and a smaller share of patients who had commercial insurance or Medicare insurance relative to NSNH.

**Table 1:** Safety-net hospital^a^ and non-safety-net hospital population summary statistics^b^

Demographics	Full Sample	SNH^c^	NSNH^d^	*p*-value^e^
		(a)	(b)	Difference a & b
Age (years)				
0–17	0.40%	0.59%	0.31%	0.000
18–29	0.66%	1.08%	0.45%	0.000
30–49	1.91%	2.88%	1.43%	0.000
50–69	15.23%	17.19%	14.25%	0.000
70 and over	81.80%	78.26%	83.57%	0.000
Gender				
Female	69.63%	67.55%	70.67%	0.000
Male	30.37%	32.45%	29.33%	0.000
Race				
Black/African American	4.77%	9.02%	2.64%	0.000
White	82.64%	70.18%	88.86%	0.000
Other	12.60%	20.80%	8.50%	0.000
Ethnicity				
Spanish/Hispanic	4.96%	9.01%	2.94%	0.000
Not Spanish/Hispanic	91.71%	85.19%	94.96%	0.000
Multiple ethnicities	0.11%	0.18%	0.08%	0.001
Other/unknown	3.22%	5.61%	2.02%	0.000
Clinical Indicator				
Length of stay (LOS) *(Days)*	6.68	7.37	6.34	0.000
Type of Admission				
Emergency, trauma, urgent	96.27%	96.17%	96.33%	0.383
Other^f^	3.73%	3.83%	3.67%	0.383
APR-DRG severity of Illness				
Minor	32.68%	30.96%	33.54%	0.000
Moderate	42.98%	43.61%	42.67%	0.050
Major	18.63%	19.46%	18.21%	0.001
Extreme	5.71%	5.97%	5.58%	0.078
APR-DRG risk of mortality				
Minor	32.15%	33.71%	31.38%	0.000
Moderate	40.37%	39.28%	40.91%	0.001
Major	21.85%	21.45%	22.05%	0.127
Extreme	5.63%	5.57%	5.66%	0.667
Hospital Indicators				
Safety-net hospital status				
Yes	33.32%			
No	66.68%			
Health insurance type				
Commercial^g^	7.05%	6.06%	7.54%	0.000
Medicaid	4.35%	7.71%	2.67%	0.000
Medicare	68.04%	54.77%	74.67%	0.000
Dual Eligible (Medicaid/Medicare)	17.01%	26.22%	12.40%	0.000
Self-pay	0.86%	1.80%	0.40%	0.000
Other/unknown^h^	2.69%	3.43%	2.32%	0.000
Disposition				
Expired	2.53%	2.47%	2.56%	0.548
Home^i^	15.72%	18.21%	14.48%	0.000
Inpatient rehabilitation facility	15.07%	18.19%	13.51%	0.000
Nursing home/long term care^j^	0.64%	0.32%	0.79%	0.000
Skilled nursing facility	61.92%	56.33%	64.72%	0.000
Other^k^	4.12%	4.48%	3.94%	0.004
N	48,948	16,310	32,638	

Note. a. safety-net hospital is defined as a hospital where at least 30% of the hospital's payer mix consists of Medicaid and uninsured payers. b. illustrated summary statistics are based on a sample of hip fracture patients from the SPARCS database with full covariate information. c. safety-net hospital. d. non-safety-net hospital. e. *p*-values from Chi-Square test or t-test examining differences between SNH and NSNH; unequal variances were adjusted for as needed. f. other admission types include: elective, newborn, and not available. g. commercial insurance includes: Blue Cross/Blue Shield; managed care, unspecific; and private health insurance. h. other insurance represents: Department of Corrections; federal/state/local/VA; miscellaneous/other; and unknown. i. patient discharged to: home or self-care; home with home health service; hospice (home); or left against medical advice. j. Medicaid Certified Nursing Home; Medicare Certified Long Term Care facility. k. patient discharged to: another facility not listed; cancer center or children’s hospital; court/law enforcement; critical access hospital; facility with custodial/supportive care; federal health care facility; hospital-based Medicare approved swing bed; hospice-medical facility; psychiatric hospital; short-term hospital

The NBRM results (Table 2) support the LOS findings from the summary statistic comparisons. Receiving hip fracture treatments at SNH was associated with a LOS estimated to be 11.7% (*p* = 0.003) longer than the estimated LOS for hip fracture treatments at NSNH. As illustrated in the Appendix (Table 4), a similar result was estimated when the sample was limited to patients aged 70 years and older. A number of demographic and clinical factors were also significantly associated with LOS. African American/Black patients were estimated to have LOS that were 20.0% (*p* < 0.001) longer than White patients, while female patients were estimated to have LOS that were 5.7% (*p* < 0.001) shorter than male patients. Additionally, patients’ APR-DRG severity of illness levels and risk of mortality levels were significantly associated with LOS; for instance, patients with moderate, major, and extreme APR-DRG severity of illness levels had LOS that were, respectively, 10.5% (*p* < 0.001), 28.6% (*p* < 0.001), and 108.6% (*p* < 0.001) longer than the estimated LOS for patients with a minor APR-DRG severity of illness level.

**Table 2:** Negative binomial regression model: Association between length of stay, hospital type,^a^ and other factors^b^

	Coefficient	Std. Error	*p*-value	95% CI	% change
Demographics					
Age (Referent: Age 70 years and over)					
0–17	− 0.54	−5.23	0.000	[− 0.74, − 0.34]	−41.7
18–29	− 0.07	− 0.98	0.328	[− 0.22, 0.07]	−7.0
30–49	− 0.06	− 1.65	0.100	[− 0.13, 0.01]	−5.6
50–69	0.04	2.66	0.008	[0.01, 0.06]	3.7
Gender (Referent: Male)					
Female	−0.06	−8.79	0.000	[−0.07, − 0.05]	−5.7
Race (Referent: White)					
Black/African American	0.18	4.16	0.000	[0.1, 0.27]	20.0
Other	0.04	1.66	0.097	[− 0.01, 0.09]	4.3
Ethnicity (Referent: Non-Spanish/Hispanic)					
Spanish/Hispanic	0.04	0.97	0.332	[−0.04, 0.11]	3.6
Multiple ethnicities	0.00	0.05	0.963	[−0.17, 0.18]	0.4
Unknown	0.00	0.11	0.909	[−0.08, 0.09]	0.5
Clinical Indicator					
Type of admission (Referent: Other^c^)					
Emergency, Trauma, Urgent	−0.47	−6.50	0.000	[−0.62, − 0.33]	−37.8
APR-DRG severity of Illness (Referent: Minor)					
Moderate	0.10	12.56	0.000	[0.08, 0.12]	10.5
Major	0.25	20.36	0.000	[0.23, 0.28]	28.6
Extreme	0.74	26.93	0.000	[0.68, 0.79]	108.9
APR-DRG risk of mortality (Referent: Minor)					
Moderate	0.12	13.79	0.000	[0.11, 0.14]	13.3
Major	0.31	23.27	0.000	[0.28, 0.33]	36.2
Extreme	0.49	19.73	0.000	[0.44, 0.54]	63.5
Hospital Indicators					
Safety-net hospital status (SNH) (Referent: No)					
Yes	0.11	2.93	0.003	[0.04, 0.18]	11.7
Health insurance type (Referent: Commerical^d^)					
Medicaid	0.22	8.34	0.000	[0.17, 0.27]	24.9
Medicare	0.01	0.88	0.376	[−0.02, 0.05]	1.5
Dual Eligible (Medicaid/Medicare)	0.03	1.11	0.266	[−0.02, 0.08]	2.9
Self-pay	−0.04	−0.78	0.437	[−0.13, 0.06]	−3.7
Other/unknown^e^	0.15	4.31	0.000	[0.08, 0.22]	16.3
Disposition (Referent: Home^f^)					
Expired	−0.33	−9.09	0.000	[−0.4, − 0.26]	−27.8
Inpatient rehabilitation facility	−0.04	−1.14	0.254	[−0.11, 0.03]	−3.9
Nursing home/long term care^g^	0.02	0.28	0.782	[−0.13, 0.17]	2.1
Skilled nursing facility	−0.01	−0.26	0.795	[−0.05, 0.04]	− 0.6
Other^h^	−0.26	−6.83	0.000	[−0.33, − 0.18]	−22.6
N	48,948				

Note. a. hospital type includes safety-net hospitals and non-safety-net hospitals where safety-net hospitals were defined as hospitals with a payer mix consisting of at least 30% of Medicaid and uninsured payers. b. illustrated statistics are coefficients and percent change amounts calculated through negative binomial regression models; the sample consisted of hip fracture patients from the SPARCS database with full covariate information; standard errors and 95% confidence intervals for the coefficients are also reported. c. other admission types include: elective, newborn, and not available. d. commercial insurance includes: Blue Cross/Blue Shield; managed care, unspecific; and private health insurance. e. other insurance in: Department of Corrections; federal/state/local/VA; other/miscellaneous; and unknown. f. patient discharged to: home or self-care; home with home health service; hospice (home); or left against medical advice. g. Medicaid Certified Nursing Home; Medicare Certified Long Term Care facility. h. patient discharged to: another facility not listed; cancer center or children’s hospital; court/law enforcement; critical access hospital; facility with custodial/supportive care; federal health care facility; hospital-based Medicare approved swing bed; hospice-medical facility; psychiatric hospital; short-term hospital

Patients’ health insurance type was also associated with LOS. Patients with Medicaid had estimated LOS that were 24.9% (*p* < 0.001) longer than patients with commercial health insurance. When the patients were stratified by the type of hospital where they received treatment, to examine whether LOS was associated with health insurance type within SNH and NSNH, respectively, the association between Medicaid and LOS remained. Within SNH, patients with Medicaid had estimated LOS that were 27.3% (*p* < 0.001) longer than patients with commercial health insurance; while within NSNH patients with Medicaid had LOS that were estimated to be 22.3% (*p* < 0.001) longer than those with commercial health insurance. Overall, the results from Tables 2 and 3 illustrate that where hip fracture patients were treated (Table 2) and their health insurance type (Table 3) were associated with their LOS.

**Table 3:** Negative binomial regression model: Association between length of stay, insurance type, and other factors^a^

	Safety-net hospitals^b^					Non-safety-net hospitals^b^				
	Coefficient	Std. Error	*p*-value	95% CI	% change	Coefficient	Std. Error	*p*-value	95% CI	% change
Demographics										
Age (Referent: Age 70 years and over)										
0–17	− 0.59	0.15	0.000	[− 0.89, − 0.29]	−44.8	−0.48	0.13	0.000	[−0.73, − 0.23]	− 38.3
18–29	− 0.13	0.08	0.121	[− 0.29, 0.03]	− 12.2	0.00	0.12	0.972	[− 0.24, 0.23]	− 0.4
30–49	− 0.04	0.04	0.298	[−0.12, 0.04]	−4.1	− 0.09	0.06	0.130	[−0.21, 0.03]	−8.6
50–69	0.05	0.02	0.029	[0.01, 0.1]	5.2	0.03	0.02	0.110	[−0.01, 0.06]	2.7
Gender (Referent: Male)										
Female	−0.06	0.01	0.000	[−0.08, − 0.04]	−5.7	−0.06	0.01	0.000	[−0.08, − 0.04]	−5.7
Race (Referent: White)										
Black/African American	0.20	0.07	0.003	[0.07, 0.33]	22.4	0.16	0.04	0.000	[0.09, 0.23]	17.3
Other	0.08	0.04	0.043	[0, 0.16]	8.4	0.00	0.03	0.942	[−0.05, 0.05]	−0.2
Ethnicity (Referent: Non-Spanish/Hispanic)										
Spanish/Hispanic	0.06	0.04	0.121	[−0.02, 0.13]	6.1	−0.01	0.05	0.762	[−0.11, 0.08]	−1.5
Multiple ethnicities	−0.02	0.13	0.867	[−0.28, 0.24]	−2.2	0.05	0.11	0.661	[−0.17, 0.27]	5.0
Unknown	−0.03	0.06	0.664	[−0.15, 0.1]	−2.7	0.06	0.05	0.230	[−0.04, 0.16]	6.3
Clinical Indicators										
Type of admission (Referent: Other^c^)										
Emergency, Trauma, Urgent	−0.54	0.11	0.000	[−0.75, − 0.33]	−41.7	−0.44	0.10	0.000	[−0.62, − 0.25]	−35.3
APR-DRG severity of Illness (Referent: Minor)										
Moderate	0.12	0.02	0.000	[0.09, 0.15]	12.4	0.09	0.01	0.000	[0.07, 0.11]	9.5
Major	0.26	0.02	0.000	[0.22, 0.31]	30.1	0.25	0.01	0.000	[0.22, 0.28]	28.0
Extreme	0.79	0.04	0.000	[0.71, 0.87]	120.3	0.71	0.04	0.000	[0.64, 0.78]	103.6
APR-DRG risk of mortality (Referent: Minor)										
Moderate	0.11	0.02	0.000	[0.08, 0.14]	11.4	0.13	0.01	0.000	[0.11, 0.16]	14.2
Major	0.28	0.02	0.000	[0.24, 0.31]	31.8	0.33	0.02	0.000	[0.29, 0.36]	38.4
Extreme	0.46	0.04	0.000	[0.38, 0.54]	58.3	0.50	0.03	0.000	[0.44, 0.57]	65.6
Hospital Indicators										
Health insurance type (Referent: Commerical^d^)										
Medicaid	0.24	0.04	0.000	[0.16, 0.33]	27.3	0.20	0.03	0.000	[0.14, 0.27]	22.3
Medicare	0.04	0.03	0.136	[−0.01, 0.1]	4.4	0.00	0.02	0.952	[−0.04, 0.04]	−0.1
Dual Eligible (Medicaid/Medicare)	0.06	0.05	0.228	[−0.04, 0.15]	6.0	0.01	0.02	0.687	[−0.04, 0.06]	1.0
Self-pay	−0.04	0.06	0.543	[−0.15, 0.08]	−3.5	−0.02	0.05	0.733	[−0.11, 0.08]	−1.6
Other/unknown^e^	0.19	0.04	0.000	[0.1, 0.28]	21.0	0.12	0.05	0.024	[0.02, 0.22]	12.3
Disposition (Referent: Home^f^)										
Expired	−0.29	0.06	0.000	[−0.42, − 0.17]	−25.3	−0.35	0.04	0.000	[−0.43, − 0.26]	−29.2
Inpatient rehabilitation facility	−0.03	0.07	0.653	[−0.17, 0.1]	−3.0	−0.05	0.03	0.150	[−0.11, 0.02]	−4.6
Nursing home/long term care^g^	0.19	0.10	0.059	[−0.01, 0.39]	21.1	−0.03	0.05	0.499	[−0.13, 0.06]	−3.3
Skilled nursing facility	−0.03	0.05	0.469	[−0.12, 0.06]	−3.3	0.01	0.02	0.720	[−0.04, 0.05]	0.8
Other^h^	−0.22	0.08	0.004	[−0.36, − 0.07]	−19.4	−0.28	0.04	0.000	[−0.36, − 0.2]	−24.6
N	16,310					32,638				

Note. a. hospital type includes safety-net hospitals and non-safety-net hospitals where safety-net hospitals were defined as hospitals with a payer mix consisting of at least 30% of Medicaid and uninsured payers. b. illustrated statistics are coefficients and percent change amounts calculated through negative binomial regression models; the sample consisted of hip fracture patients from the SPARCS database with full covariate information; standard errors and 95% confidence intervals for the coefficients are also reported. c. other types of admission include: elective, newborn, and not available. d. commercial insurance includes: Blue Cross/Blue Shield; managed care, unspecific; and private health insurance. e. other insurance represents: Department of Corrections; federal/state/local/VA; other/miscellaneous; and unknown. f. patient discharged to: home or self-care; home with home health service; hospice (home); or left against medical advice. g. Medicaid Certified Nursing Home; Medicare Certified Long Term Care facility. h. patient discharged to: another facility not listed; cancer center or children’s hospital; court/law enforcement; critical access hospital; facility with custodial/supportive care; federal health care facility; hospital-based Medicare approved swing bed; hospice-medical facility; psychiatric hospital; short-term hospital

## Discussion

Studies have estimated disparities by race and/or ethnicity in health care outcomes in general [42, 43] as well as more specifically for hip-fracture [8] outcomes (e.g., readmissions) [9] and treatments (e.g., discharged to rehabilitation) [7, 44]. The results of this study illustrate that disparities in hip fracture treatment outcomes may arise not only from demographic factors, but also from the type of hospital where patients received treatments as well as their health insurance type. The average LOS for hip fracture treatments was statistically higher in New York State SNH relative to New York State NSNH; and receiving hip fracture treatments in a SNH was associated with a longer LOS relative to receiving treatment in an NSNH. Furthermore, relative to commercial health insurance, a positive association was estimated between Medicaid and LOS for hip fracture treatments. While the study findings are based upon associations, which do not imply causation, the results are meaningful; they illustrate that in addition to social factors, disparities in hip fracture treatment outcomes may be partially due to where patients receive treatment and how they pay for their treatments.

The estimated LOS differences between SNH and NSNH may result in poorer health outcomes for hip fracture patients treated at SNH relative to those treated at NSNH. Nikkel et al. [33], using a sample of 169,258 patients from the New York State SPARCS dataset, found that shorter LOS for hip fracture treatments was associated with reduced rates of mortality. The Nikkel et al. result conflicted with two European studies that found longer LOS were associated with decreased risk of mortality [31, 32]. Nikkel et al. (2015) argue that the contradiction could be due to differences in treatment structures (e.g., time management of hip fracture treatments) or payment systems (e.g. financial considerations in the U.S. that might yield a more rapid discharge), concluding that caution should be used when comparing results from different health care systems [33]. The differences in hip fracture treatment LOS found here illustrate that treatment structures and payment systems may not only explain differences across nations, but differences between hospital types within nations as well.

In this study (Table 1), as well as others [24, 45], the demographic characteristics of patients treated in SNH and NSNH statistically varied, which may help explain some of the LOS difference between these hospital types for hip fracture treatments. For instance, based upon demographic characteristics, SNH providers may extend patients LOS to account for a perception that their patients have insufficient social support upon discharge [7] or to provide patients with inadequate access to rehabilitation services with similar inpatient services (e.g. acute care rehabilitation). However, Harda, Chun, and Chiu estimated that patients in disproportionate share hospitals were less likely to receive acute care physical therapy after hip fracture treatments relative to patients receiving care in non-disproportionate share hospitals [44]; a result that supports the concept that the treatment patients receive and their subsequent health outcomes may differ on components beyond demographic characteristics, such as hospital based-factors [16, 17, 24, 25]. Other patient-based variables, such as health insurance type, were also associated with LOS both within and between hospital categories. The LOS differences based upon health insurance type within a SNH or a NSNH entails that even if LOS were similar between SNH and NSNH for hip fracture treatments, that patients’ health insurance type could result in different outcomes within the same category of hospital (SNH, NSNH).

While it is important to note that patients’ health insurance types may help determine LOS for hip fracture treatments, the results of this study illustrate that patient-based variables alone do not explain LOS differences between SNH and NSNH. After controlling for age, gender, race, ethnicity, and health insurance type a significant, positive association remained between LOS and receiving hip fracture treatment in a SNH relative to receiving treatment in a NSNH (Table 2). Similarly, the results illustrate that patient health characteristics do not fully explain the LOS differences between SNH and NSNH. Patient comorbidities were not directly measured in this study, which is a limitation; however, the influence of comorbidities on LOS may be partially captured through the APR-DRG severity of illness levels (SOI) and the APR-DRG risk of mortality levels (ROM) in the NBRM models. Secondary diagnoses are one component in the calculation for the APR-DRG SOI and ROM levels. Yet, in the presence of the APR-DRG SOI and ROM controls, LOS remained positively associated with being treated in a SNH relative to a NSNH.

The APR-DRG measures, while indicating the health status of patients, do not fully capture the influence of hip fracture treatment complications or processes on LOS, which is also a limitation. However, treatment complications could arise from overall structural differences in SNH and NSNH treatment processes (e.g., time to surgery [13]). For instance, differences in treatment processes could arise if SNH lack the resources to implement effective surgical treatment protocols and/or organizational structures that may improve patient outcomes [8]. Indeed, the payer mix of SNH (e.g., a mix with a large share of Medicaid payers) may negatively influence the financial condition of SNH [21], resulting in less available resources and thereby influencing patient outcomes. Accordingly, an underinvestment in protocols intended to increase quality could explain the extended LOS for hip fracture treatments in SNH, relative to the LOS for hip fracture treatments in NSNH. The pertinent question is not whether time to surgery varied between SNH and NSNH, but, whether this variation is due to different resources available in SNH relative to NSNH.

A conclusion is not readily available from the present research regarding the direct role of payer mix on hospital resources and hip fracture outcomes. However, from a statistical association perspective (which does not imply causation), the results illustrate that hip fracture treatment outcomes, after controlling for socioeconomic factors, differ between SNH and NSNH. Accordingly, hospital-based factors (e.g., different treatment programs, investments in quality initiatives and/or technology) may also play a role in outcome disparities between groups. Assuming these hospital-based factors are associated with resource availability, decreasing disproportionate hospital share payments or penalizing hospitals that do not meet quality metrics under a value-based payment system may further decrease revenues for SNH [24, 46], which could in turn increase disparities in health care outcomes between patients receiving care in SNH relative to NSNH.

The association between the financial condition of hospitals and hip fracture treatment outcomes was not evaluated here which is a study limitation; future research should evaluate the relationship between hip fracture treatment outcomes, hospital financial condition, hospital type, and hospital-based treatment procedures. Beyond the comorbidity and complexity limitations highlighted in the text, an additional study limitation was the omission of treatment protocols and other clinical and patient level factors that may influence LOS. Selection bias may also be present if patients selected the hospital for their treatment; this could result in differing case-mixes and risk profiles between the hospital types. However, the models controlled for APR-DRG severity of illness and risk of mortality levels and the majority of patients were admitted to hospitals through an “emergency/trauma/urgent” setting, which likely limited the ability of patients to select where their hip fracture treatments occurred. Regardless, if selection bias is present it could influence LOS. Allowing age to enter the model in age groups, instead of as a continuous variable, was also a limitation; one that was due to the data available. As a final limitation, it should be noted that the regression results are correlations and do not imply causation.

## Conclusion

Length of stay (LOS) is an important quality indicator for hip fracture treatments. Results of this study illustrate that patients treated for hip fractures in safety-net hospitals have extended LOS relative to patients treated in non-safety-net hospitals; a difference that may result in adverse health outcomes for those treated in safety-net hospitals. The difference in LOS between safety-net and non-safety-net hospitals remained significant after controlling for patient demographic and clinical characteristics, suggesting that hospital-based factors might help explain the longer LOS in safety-net hospitals relative to non-safety-net hospitals. Future research should evaluate the role of hospital-based factors such as hospital resources and payer mix in hip fracture treatment patient outcomes. Such research may help inform policy solutions to address quality of care challenges in hospitals as well as health care outcome disparities between patients treated at safety-net and those treated at non-safety-net hospitals.

## Data Availability

The datasets generated and/or analyzed during the current study are available from the New York State Department of Health Statewide Planning and Research Cooperative System (SPARCS), https://www.health.ny.gov/statistics/sparcs/.

## Appendix

**Table 4:** Negative binomial regression model: Association between length of stay, hospital type,^a^ and other factors among respondents aged 70 years and older^b^

	Coefficient	Std. Error	*p*-value	95% CI	% change
Demographics					
Gender (Referent: Male)					
Female	−0.06	0.01	0.00	[−0.07, − 0.04]	− 5.7
Race (Referent: White)					
Black/African American	0.18	0.05	0.00	[0.08, 0.27]	19.2
Other	0.03	0.03	0.22	[−0.02, 0.08]	3.1
Ethnicity (Referent: Non-Spanish/Hispanic)					
Spanish/Hispanic	0.03	0.03	0.34	[−0.03, 0.1]	3.3
Multiple ethnicities	0.07	0.10	0.53	[−0.14, 0.27]	6.7
Unknown	−0.01	0.04	0.86	[−0.09, 0.07]	−0.7
Clinical Indicator					
Type of admission (Referent: Other^c^)					
Emergency, Trauma, Urgent	−0.51	0.07	0.00	[−0.64, − 0.39]	−40.1
APR-DRG severity of Illness (Referent: Minor)					
Moderate	0.08	0.01	0.00	[0.07, 0.09]	8.3
Major	0.21	0.01	0.00	[0.18, 0.23]	22.9
Extreme	0.64	0.03	0.00	[0.58, 0.69]	89
APR-DRG risk of mortality (Referent: Minor)					
Moderate	0.10	0.01	0.00	[0.09, 0.12]	10.8
Major	0.31	0.01	0.00	[0.28, 0.33]	36.2
Extreme	0.53	0.03	0.00	[0.48, 0.59]	70.6
Hospital Indicators					
Safety-net hospital status (SNH) (Referent: No)					
Yes	0.10	0.04	0.01	[0.03, 0.18]	10.9
Health insurance type (Referent: Commerical^d^)					
Medicaid	0.17	0.05	0.00	[0.08, 0.27]	18.6
Medicare	−0.04	0.03	0.10	[− 0.09, 0.01]	−4.1
Dual-eligible (Medicaid/Medicare)	−0.03	0.03	0.31	[−0.1, 0.03]	−3.3
Self-pay	−0.06	0.06	0.35	[−0.17, 0.06]	−5.4
Other/unknown^e^	0.07	0.05	0.16	[−0.03, 0.17]	7.5
Disposition (Referent: Home^f^)					
Expired	−0.42	0.03	0.00	[−0.48, − 0.35]	−34.1
Inpatient rehabilitation facility	−0.13	0.04	0.00	[−0.2, − 0.05]	−11.9
Nursing home/long term care^g^	−0.10	0.07	0.15	[−0.24, 0.04]	−9.6
Skilled nursing facility	−0.09	0.02	0.00	[−0.14, − 0.05]	−8.9
Other^h^	−0.36	0.04	0.00	[−0.43, − 0.28]	−29.9
N	40,039				

Note. a. hospital type includes safety-net hospitals and non-safety-net hospitals where safety-net hospitals were defined as hospitals with a payer mix consisting of at least 30% of Medicaid and uninsured payers. b. illustrated statistics are coefficients and percent change amounts calculated through negative binomial regression models; the sample consisted of hip fracture patients from the SPARCS database with full covariate information; standard errors and 95% confidence intervals for the coefficients are also reported. c. other admission types of include: elective, newborn, and not available. d. commercial insurance includes: Blue Cross/Blue Shield; managed care, unspecific; and private health insurance. e. other insurance includes: Department of Corrections; federal/state/local/VA; other/miscellaneous; and unknown. f. patient discharged to: home or self-care; home with home health service; hospice (home); or left against medical advice. g. Medicaid Certified Nursing Home; Medicare Certified Long Term Care facility. h. patient discharged to: another facility not listed; cancer center or children’s hospital; court/law enforcement; critical access hospital; facility with custodial/supportive care; federal health care facility; hospital-based Medicare approved swing bed; hospice-medical facility; psychiatric hospital; short-term hospital

## Abbreviations

APR-DRG: All Patient Refined Diagnostic Related Groups
CCS: Clinical Classification Software diagnosis code
DSH: Medicaid disproportionate share payments
DSRIP: New York State’s Delivery System Reform Incentive Payment Program
ICD: International Classification of Diseases
LOS: Length of stay
NBRM: Negative binominal regression models
NSNH: Non-safety-net-hospitals
ROM: APR-DRG risk of mortality levels (ROM)
SNH: Safety-net hospitals
SOI: APR-DRG severity of illness levels
SPARCS: New York State’s Statewide Planning and Research Cooperative System
